# An analysis of farmers' perception of the new cooperative medical system in Liaoning Province, China

**DOI:** 10.1186/1472-6963-9-230

**Published:** 2009-12-12

**Authors:** Xilong Pan, Ying Zhang, Li Xu, Ju Huang, Qianqian Zhao

**Affiliations:** 1 Peking University Health Science Center, School of Public Health, Health Policy and Management Department, No.38 Xue Yuan Road, Haidian District, Beijing 100191, PR China

## Abstract

**Background:**

Since 2003, the number of pilot areas of the New Rural Cooperative Medical System (NRCMS) has increased in rural China. And the major efforts have been concentrated on the enrollment of prospective members. In this study, we examined the satisfaction of the rural residents with the NRCMS as well as factors affecting their attitudes towards the NRCMS.

**Methods:**

The data for this study were collected from a survey involving twenty counties in Liaoning Province. Interviews and focus groups were conducted between 10^th ^January and 20^th ^August 2008. A total of 2,780 people aged 18-72 were randomly selected and interviewed. Data were evaluated by nonparametric tests and ordinal regression models.

**Results:**

71.6% of the study subjects were satisfied with the NRCMS. Single factor analysis showed that attitudes towards the NRCMS were influenced by gender, age, marital status, and self-rated health status. In the ordinal regression analysis, gender, age, and self-rated health status affect satisfaction (P < 0.05).

**Conclusions:**

We found that a considerable proportion of farmers were satisfied with the NRCMS. Gender, age, and self-rated health status had significant effects on farmers' attitudes towards the NRCMS. The Chinese Central Government attempted to adopt active measures in the future to continuously improve the NRCMS, including initiating educational programs, building new medical facilities and increasing financial investment.

## Background

The New Cooperative Medical System (NCMS) is a main component of the Chinese new rural medical insurance system. The development of Chinese rural medical insurance system has gone through three stages:

The forming period of the Cooperative Medical System (CMS) (1958-1980s): Before 1958, economic development was slow in rural China. No medical insurance system existed. In 1958, the National People's Congress of China (NPC) decided that the rural cooperative community, which was based on traditional geographic village, should be built as the most fundamental government agency in rural areas,. The CMS was established in the rural cooperative community to provide basic medical insurance to the community memembers, and in return each member made fixed contribution to CMS every year. Thus the communitymembers were insured by the CMS in case they needed medical care. This system played a significant role in promoting healthcare in rural China. Till the end of 1979, about 90% of administrative villages in China implemented the CMS. The reform and opening up of China changed every aspect of the Chinese society. In the late 1970s, with the collapse of the collective economic power of the rural cooperative communities, the CMS, which mainly depended on collective funding from cooperative communities, disintegrated. In 1985, the CMS could be found in only five percent of administrative villages.

The recession period of the Rural Cooperative Medical System (RCMS) (1990s-2001): Till the early 1990s, the RCMS only existed in villages in Shanghai and southern Jiangsu Provinces still had [1]. In 1997, the Chinese Central Government started the Rural Cooperative Medical System (RCMS). The system followed the voluntary principle and relied heavily on the contributions of the villages involved and the individual participating farmers. The RCMS premium for each participant was less than 10 Yuan (about 1.5 US dollars) per year. At the same time, the Central Government provided additional 10 Yuan subsidies to each participating farmer who lived in less developed areas, most of which were in central and western regions. The development period of the New Rural Cooperative Medical System (NRCMS) (since 2002): In 2002, the central government noticed the increasing medical costs and thus decided to provide additional financial assistance to help those who lived below the poverty line. While the government continues to emphasize that the premium is individual's responsibility, the policy made useful extension and pave the way for further development [2]. Compared to the RCMS, the NRCMS had the following characteristics: (1) adoption of principles and methods of modern insurance system; new fundraising, modern compensation and payment mechanisms; (2) voluntary participation; (3) broader coverage; and (4) different implementation with local conditions. The NRCMS was launched in 2,451 rural areas, accounting for 86% of the total, involving 730 million people till the end of 2007. The real funding per capita was 58.8 Yuan and 42.8 billion Yuan in total in 2007. The central government set a goal to extend the coverage of the NCMS to all around China by 2010[3].

Current literatures have focused on enrollment issues, applied functions, and various challenges associated with the NCMS [4-7]. Evidence indicates that poor rural residents are unable to afford the NCMS even though the premium is low [8]. Researches also suggest that reimbursements of extensive medical care (i.e., serious illness) only cover about 20-60% of total inpatient costs and 10-30% of outpatient costs [9,10]. Many questions remain unanswered: Do rural people know the existence of the NCMS and the relevant policies? Do they understand the coverage of the NCMS? What factors affect the enrollment rate? Are beneficiaries satisfied with the NCRMS and are they willing to continue? [11-13]

In this paper, we intend to find the answers to the questions above. Compared to the sample Weihai, a small city in China, surveyed in a previous study [14,15], this study selects Liaoning Province. This appears to be a more representative sample with not only larger sample size but also developed and less developed areas involved. Tthe findings of the study help to understand the performance of the NCMS, the sustainability and future improvements.

## Methods

### Study sample

The sample of both focus groups and questionnaires was collected in Liaoning Province, including both developed areas and less developed areas. In 2007, the poverty line in Liaoning Province was defined as 2,000 Yuan, while the average per capita income of the province was 4,773 Yuan. Areas with average income per capita above (below) 2,000 Yuan were regarded as developed (less developed) areas. In our sample 10 villages were randomly selected in each developed and less developed areas respectively and 3,000 people aged 18-72 were involved. Both in-home questionnaire and additional focus groups were conducted between 10th January and 20th August 2008 [16].

In a typical interview, one expert held the interview and two professionals recorded the conversation, expression and mood of the interviewees throughout the process [17]. Informed consent was obtained from the participants before the interview^1^.

### Data collection and measures

All participants completed a structured questionnaire. The following items were included: socio-demographics(gender, age, education level, marital status), self-rated health status, whether being sick within 2 weeks, and attitudes towards the NCMS. Initially, items of attitudes towards the NCMS consisted of five options: "Very Satisfied" "Satisfied", "Neutral", "Dissatisfied", and "Very Dissatisfied". Our initial descriptive analysis indicated that answers to each extreme were rare. We, therefore, combined "Very Satisfied" and "Satisfied" into "Satisfied". "Dissatisfied" and "very dissatisfied" were integrated into "dissatisfied". In the original questionnaire, occupation was included. We excluded it as all participants were farmers.

### Statistical analysis

There were two sections in the questionnaire: (1) merits of the NCMS (7 items); (2) drawbacks of the NCMS (8 items). The respondents could select one or more items based on their own perception and experience. To be consistent in comparison, each item was converted to percentage using the following formula:(1)

Where A = percentage of a particular item

Ω = number of respondents selected the item;

k = total number of valid respondents

In order to investigate factors affecting overall satisfaction, we conducted nonparametric tests to compare differences in attitudes towards the NCMS among respondents with different socio-demographic attributes, self-rated health status and whether being sick within two weeks. We applied ordinal regression models to evaluate effects of factors on attitudes towards the NCMS [18-20]. In the ordinal regression models, dependent variable Y, the respondents' satisfaction of the NCMS was divided into 3 categories (1 as satisfied, 2 as Neutral, 3 as Dissatisfied). The results of nonparametric analysis were substituted into the equation as follows:

The regression equation is:(2)

where γj(x) = π_1_(x)+π_2_(x)+...+π_j_(x), θ_j _= logk_j_

X_1 _= Gender (male = 1, female = 0); X_2 _= Age (younger than 20, X_2 _= 0; 20-40, X_2 _= 1; 40-60, X_2 _= 2; X_2 _= 3, older than 60);

X_3 _= marital status (Single = 0, Married = 1, Divorced or widowed = 2);

X_4 _= self-rated health status (Excellent = 0, Good = 1, Poor = 2, Awful = 3).

The study was approved by Peking University Health Science Center Institutional Review Board (No: IRB00001052-T1) and had no violation to human subjects, privacy and community honor.

## Results

A total of 3,000 questionnaires were distributed. 2,780 questionnaires (92.66%) were returned. 26 questionnaires were not fully answered and considered void. In total, 2,754 questionnaires (91.80%) were included in this study.

The basic characteristics of study respondents and their attitudes towards the NCMS were presented in Table 1. Of the study population, 71.6% were satisfied with the NCMS, 5.3% were not satisfied, and 23.1% expressed neutral attitudes. When asked about the main reason resulting in the dissatisfaction with the NCMS, 70.6% of 782 respondents referred to the low reimbursement rate, while 19.7% complained about poor quality of care, and 9.7% thought other benefits of the NCMS should be included in the future.

**Table 1:** Factors associated with attitudes towards NCMS

Variables	Satisfaction (%)	intermediate (%)	dissatisfaction (%)	t-value	p
Gender				-2.78	0.005

Male	76.9	19.5	3.6		

Female	67.2	24.3	8.5		

Age(years)				23.59	0.000

<20	63.8	27.4	8.8		

20~40	62.9	26.7	10.4		

40~60	74.6	19.6	5.8		

>60	80.4	13.8	5.8		

Education level				6.73	0.187

Illiterate	74.5	18.0	7.5		

Elementary school	70.7	22.4	6.9		

Junior high school	70.8	22.9	6.3		

High school graduates or above	75.2	20.4	4.4		

Marital status				19.77	0.000

Single	70.5	20.8	8.7		

Married	77.4	17.3	5.2		

Divorced or widowed	70.9	24.7	4.4		

Self-rated health status				17.69	0.000

Excellent	83.2	13.7	3.1		

Good	74.5	18.6	6.9		

Poor	70.3	24.3	7.4		

Awful	63.7	25.9	10.4		

Sick within latest two weeks				-1.86	0.079

Yes	70.2	25.4	4.4		

No	72.4	21.9	5.7		

**Figure 1:**
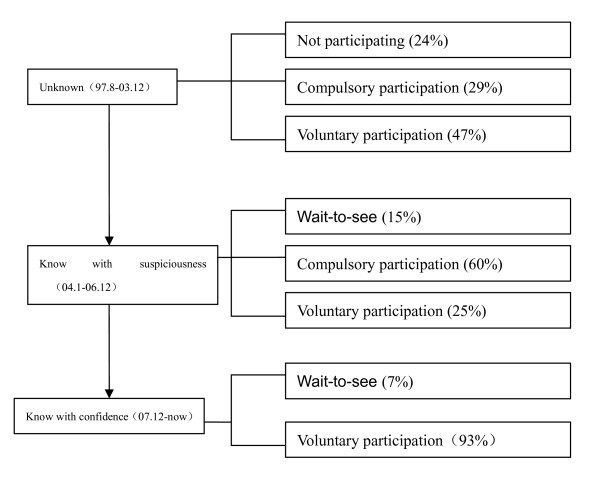
**Changes of rural residents' view on NCMS**.

Among the study respondents, the ratio of male to female was 0.89 (1263/1491). 76.9% of men and 67.2% of women were satisfied with the NCMS. The difference between men and women was statistically significant (P < 0.05). The difference was also statistically significant among those of different age groups (P < 0.05). This difference mostly resulted from the 20~40 group (62.9% satisfied) and the >60 group (80.4%, satisfied). Statistically significant difference was found among those of different marital status (P < 0.05). Regarding self-rated health status, those with excellent health had highest satisfaction rate (83.2%). 74.5% with good health were satisfied. So were 70.3% in those with poor health, and 63.7% with awful health. The difference was statistically significant (P < 0.05). Satisfaction rate of the NCMS was unaffected by education level and whether being sick within two weeks in this study. (Table 1, Figure 1)

The Study respondents widely acknowledged the NCMS benefits, such as coverage for major diseases, coverage cap, deductibles and compensation rates. They were worried about low compensation rate and poor quality of care. (Table 2)

**Table 2:** Survey of agreement and anxiety of NCMS

Item	Percent(%)	ranking
Agreement of NCMS		

Government provides fiscal subsidies	30.8	3

Medical fee is reimbursed	52.3	2

Treatment is adequate	6.8	4

Major disease is covered	90.2	1

Physician offices are close to home	2.6	7

Avoid the doctors raise their rates	3.3	6

Unknown (what is that)	4.4	5

Anxiety about NCMS		

Farmers have to pay even if they don't use service	1.5	7

Reimbursement rate is too low	25.8	2

Claiming procedures are tedious	14.3	4

Care is not free	7.5	6

Medicine is ineffective	0.7	8

Quality of care is poor	16.7	3

Costs on medicine are increasing	11.1	5

Policies and Rules are unclear	77.7	1

Ordinal regression model was adopted to test the satisfaction from patients towards the NCRMS. According to the basic assumption of ordinal regression model (Independent variable should contain three or more categories. One or more explanatory variables are included in this model.), the data from our survey were suitable to be conducted in this model. The ordinal regression analysis revealed that gender, age and self-rated health status had large impacts on attitudes towards the NCRMS. However, influences of marital status were insignificant.

## Discussion

### (1) Analysis of three factors

This study found 71.6% of study respondents were satisfied with the NCMS and identified that three factors (gender, age and self-rated health status) were significantly associated with satisfaction.

Gender significantly affects attitudes towards the NCMS. 76.9% of men were satisfied, whereas 67.2% of women were satisfied. Through typical interviews, we understood that men provided the main source of household income, therefore they were engaged in agricultural work as the main labor force in rural areas. However, women were mainly responsible for family daily life. Therefore, the NCMS did not provide immediate benefits to women [21]. Hence the men had a higher rate of recognition and satisfaction with the NCMS.

The study showed that as people got older, satisfaction rate also rose (except the 20-40 age group). This could be explained by the fact that people between 20 and 40 were more economically independent than those in other age groups. The elderly were more likely to be satisfied with the current system since the elderly had the most medical needs, and expected government supports the most, especially when a welfare support system has been available for a long time [22,23]. Through typical interviews, we understood that as long as the elderly were able to afford the premium and co-payments, they were more likely to be satisfied with the NCMS. It was also not surprising that individuals who thought medical care was expensive tended to be less satisfied with the overall performance of the NCMS. Individuals who were satisfied with the reimbursement procedure of the NCMS were more likely to be satisfied with the overall performance of the NCMS.

Furthermore, self-rated health status played an indispensable role regarding the attitudes towards the NCMS. Compared to those of excellent self-rated health status, respondents with awful self-rated health had lower satisfaction rate. One explanation might be that the NCMS could not completely meet the needs of persons who were in a poor health condition [24]. Through typical interviews, we understood that though the NCMS could, to some extent, ease patients' economic burdens, it was not enough to guarantee patients, especially those with catastrophic diseases, to get suitable and timely care without running into financial difficulty. At the same time, hospital expenses were usually reimbursed after patients had paid the full medical costs. So if a patient failed to afford the cost in the first place, he could not even get the necessary medical treatment, not even to mention the reimbursements from the NCMS. The situation was made even worse when those who could not pay the cost in advance were actually those need the NCMS the most. If a certain proportion of expense could be exempted in advance, we anticipated that satisfaction rate would increase.

### (2) Farmers ' perception of the NCMS

When the NCMS was implemented in 2003, most farmers did not know the NCMS and they did not want to participate in the NCMS. Some local government officials for political reasons forced farmers to take part in the NCMS. In recent years, the number of farmers who voluntarily participate in the NCMS has increased, though some people still take a wait-and-see attitude [25].

The reason of the climbing participation rate was partly due to the increased governmental subsidies. During the in-depth interviews, respondents generally recognized the NCMS policy, even though 77.71% of respondents did not know the detailed regulations of the NCMS. (Table 2).

The study showed that the NCRMS provided inadequate protection against major diseases. Small amount of premium and low government subsidies caused narrow coverage and small reimbursement rate. Once farmers suffered from serious disease and could not afford the medical costs, they would most likely to abandon treatment.

An issue worth emphasizing is that service ability of grass-roots hospitals should be strengthened. Due to long-term insufficient government investments, the infrastructure of rural clinics and township health centers were far from adequate. Medical facilities and equipments were simple. Physicians were lack of proper medical trainings. The medical infrastructure could not meet farmers' medical needs. The quality of care was not warranted.

### (3) Policy suggestions

Since the start of the NCMS, more farmers have acknowledged the NCMS and are willing to participate in the NCMS. They know that NCMS policies could bring real benefits. The NCMS would most likely to be successful in the long run with the continuous improvement and more benefits available to farmers.

By 2008, the Chinese government modified fundraising structure and raised reimbursement rate in order to expand the coverage of the NCMS. In the new structure, farmers' premium increased from 10 Yuan to 20 Yuan per year. The governmental subsidies raised to 80 Yuan per capita per year.

Due to asymmetric information of medical care, farmers are in a disadvantaged position; medical care is dominated by physicians and medical institutions. Under the NCMS, medical institutions may tend to provide unnecessary treatments to the patients. Therefore, the administration of the NCMS should be strengthened. The reform of healthcare system must be modified accordingly, including increasing health investments, optimizing health resources allocation, improving qualities of medical care, developing drug delivery system and pricing mechanism in rural China. Medical institutions should adapt themselves to meet the demands of the NCMS and improve medical practice to ensure the quality of care, and protect farmers' legitimate rights.

In rural China, insufficient supply of physicians and inactive adoption of high technology are mainly caused by low incomes of medical staffs and rural medical institutions. As a result, medical staffs with extensive medical trainings are unwilling to work there. State funding should be devoted in rural clinics and township (Commune) health centers, and physicians training should be strengthened.

In order to improve the NCMS steadily and soundly, explicit policies and rules of the NCMS should be established as soon as possible. All these can clarify governments' and medical institutions' responsibility, and provide enough protection for insured farmers. The NCMS shall be ruled, implemented and administrated by law.

This study has one limitation. More women than expected are involved in the sample of this study as more women than men were available in rural areas when the survey was conducted.

## Conclusions

In summary, we found that a considerable proportion of farmers were satisfied with the NCMS. In addition, gender, age, and self-rated health status have large contribution to farmers' attitudes towards the NCMS. These findings will help the chinese central and local governments make future plans to improve the NCMS.

In recent years, the central and local governments have adopted effective measures to improve medical trainings, state health finance and the construction of the NRCMS. The NRCMS still faces lots of challenges, such as poor management of medical institutions in villages and townships, low standard for the medicine catalogues, lack of compensation for severe diseases, insufficient care for peasant workers, complicated claiming procedures and financing of the NCMS. Clearly, until these challenges are successfully met, health care problem of one billion farmers will be alleviated. This is a medical problem, also a social problem, and requires serious governmental consideration at all levels.

## Abbreviations

RCMS: Rural Cooperative Medical System; NRCMS: New Rural Cooperative Medical System; NPC: National People's Congress of China.

## Competing interests

The authors declare that they have no competing interests.

## Authors' contributions

XLP designed and supervised the study, and provided statistical advice throughout the study, YZ analyzed and interpreted data, and drafted the manuscript and finalized writing it, ZW revised the article, others involved in performing the surveys, collected data from the Liaoning Province. All authors read and approved the final version of the manuscript.

## Pre-publication history

The pre-publication history for this paper can be accessed here:

http://www.biomedcentral.com/1472-6963/9/230/prepub
